# Adipocytes WNT5a mediated dedifferentiation: a possible target in pancreatic cancer microenvironment

**DOI:** 10.18632/oncotarget.7936

**Published:** 2016-03-06

**Authors:** Elena Zoico, Elena Darra, Vanni Rizzatti, Simona Budui, Guido Franceschetti, Gloria Mazzali, Andrea P Rossi, Francesco Fantin, Marta Menegazzi, Saverio Cinti, Mauro Zamboni

**Affiliations:** ^1^ Department of Medicine, Geriatrics Section, University of Verona, Verona, Italy; ^2^ Department of Life and Reproduction Sciences, Biochemistry Section, University of Verona, Verona, Italy; ^3^ Department of Experimental and Clinical Medicine, Center of Obesity-University of Ancona (Politecnica delle Marche), Ancona, Italy

**Keywords:** pancreatic cancer, adipocytes, tumor microenvironment, cell dedifferentiation, WNT5a

## Abstract

A significant epidemiological association between obesity and pancreatic ductal adenocarcinoma (PDAC) has previously been described, as well as a correlation between the degree of pancreatic steatosis, PDAC risk and prognosis. The underlying mechanisms are still not completely known.

After co-culture of 3T3-L1 adipocytes and MiaPaCa2 with an *in vitro* transwell system we observed the appearance of fibroblast-like cells, along with a decrease in number and size of remaining adipocytes. RT-PCR analyses of 3T3-L1 adipocytes in co-culture showed a decrease in gene expression of typical markers of mature adipocytes, in parallel with an increased expression of fibroblast-specific and reprogramming genes. We found an increased WNT5a gene and protein expression early in MiaPaCa2 cells in co-culture. Additionally, EMSA of c-Jun and AP1 in 3T3-L1 demonstrated an increased activation in adipocytes after co-culture. Treatment with WNT5a neutralizing antibody completely reverted the activation of c-Jun and AP1 observed in co-cultured adipocytes.

Increasing doses of recombinant SFRP-5, a competitive inhibitor for WNT5a receptor, added to the co-culture medium, were able to block the dedifferentiation of adipocytes in co-culture.

These data support a WNT5a-mediated dedifferentiation process with adipocytes reprogramming toward fibroblast-like cells that might profoundly influence cancer microenvironment.

## INTRODUCTION

Obesity has already been demonstrated from an epidemiological point of view to be an independent risk factor for pancreatic ductal adenocarcinoma (PDAC) [1–3]. A significant association between the degree of histologically determined pancreatic steatosis, and risk of PDAC has recently been described [4, 5], with increased pancreatic fat also representing a prognostic negative marker for PDAC patients [6]. However the mechanisms underlying the link between pancreatic cancer, obesity and pancreatic steatosis are still not completely understood. A PDAC tumor microenvironment comprises several different cell types and is mainly characterized by a desmoplastic fibrotic reaction whose origin is not completely known [8].

Adipose tissue (AT) has been recognized as an important constituent of cancer microenvironment in some types of tumors [9, 10]. AT has been regarded as an endocrine organ characterized by high physiological plasticity [11] as it has been shown that even mature fat cells are able to dedifferentiate generating cells with multipotent capacities [12, 13]. Moreover obesity has been considered as a state of chronic low grade inflammation where AT is responsible for an altered production of pro-inflammatory and chemoattractant chemokines [14, 15]. More importantly, recent data concerning esophageal adenocarcinoma (EAC) suggested that peritumoral AT exerted a direct effect on disease progression, mainly by secreting depot-specific paracrine factors, such as leptin [16].

At a biomolecular level, it has been reported that circulating WNT5a concentration is increased in human obesity [17] and that noncanonical Wnt signaling is activated, probably contributing to the pro-inflammatory state in visceral adipose tissue. Some studies identified a crucial role for WNT5a also in cancer progression and metastatization [18] with conflicting results in different types of cancer [19]. In pancreatic cancer, WNT5a is recurrently over-expressed and exerts a pro-oncogenic function by promoting proliferation, migration and invasion of cancer cells [20–24]. This up-regulation of WNT5a in PDAC models has been recently shown to be involved also in epithelial-to-mesenchymal transition [20, 25]. Moreover, in pancreatic cancer cells, an activation of the JAK-STAT3 pathway that could drive WNT5a activation, has been shown to be induced by inflammatory cytokines produced also by AT [26, 27].

In order to study the existence of a crosstalk between adipocytes and pancreatic cancer cells and its consequences in a tumor microenvironment, we set up an *in vitro* transwell system where 3T3-L1 adipocytes were co-cultured with MiaPaCa2 cells and analyzed for morphological and functional changes [28]. Our data support the existence of a process characterized by adipocytes dedifferentiation/reprogramming toward fibroblasts-like cells mediated by the WNT5a pathway.

## RESULTS

### Mature 3T3-L1 adipocytes dedifferentiate to fibroblast-like cells after co-culture with MiaPaCa2

In order to study the crosstalk between adipocytes and pancreatic cancer cells, we used a co-culture model where MiaPaCa2 cells were seeded in the top chamber and 3T3-L1 in the bottom of a transwell culture system, starting 5 days after adipocyte induction (post induction day = PID 5) and maintained in co-culture for 3 (PID 8), 6 (PID 11) and 9 (PID 14) days. 3T3-L1 adipocytes cells were cultivated alone and studied at the same time points as controls.

Vitality of cells under the different experimental conditions was assayed by trypan blue and did not significantly change at the different time points between co-culture and control adipocytes (data not shown).

**Figure 1:**
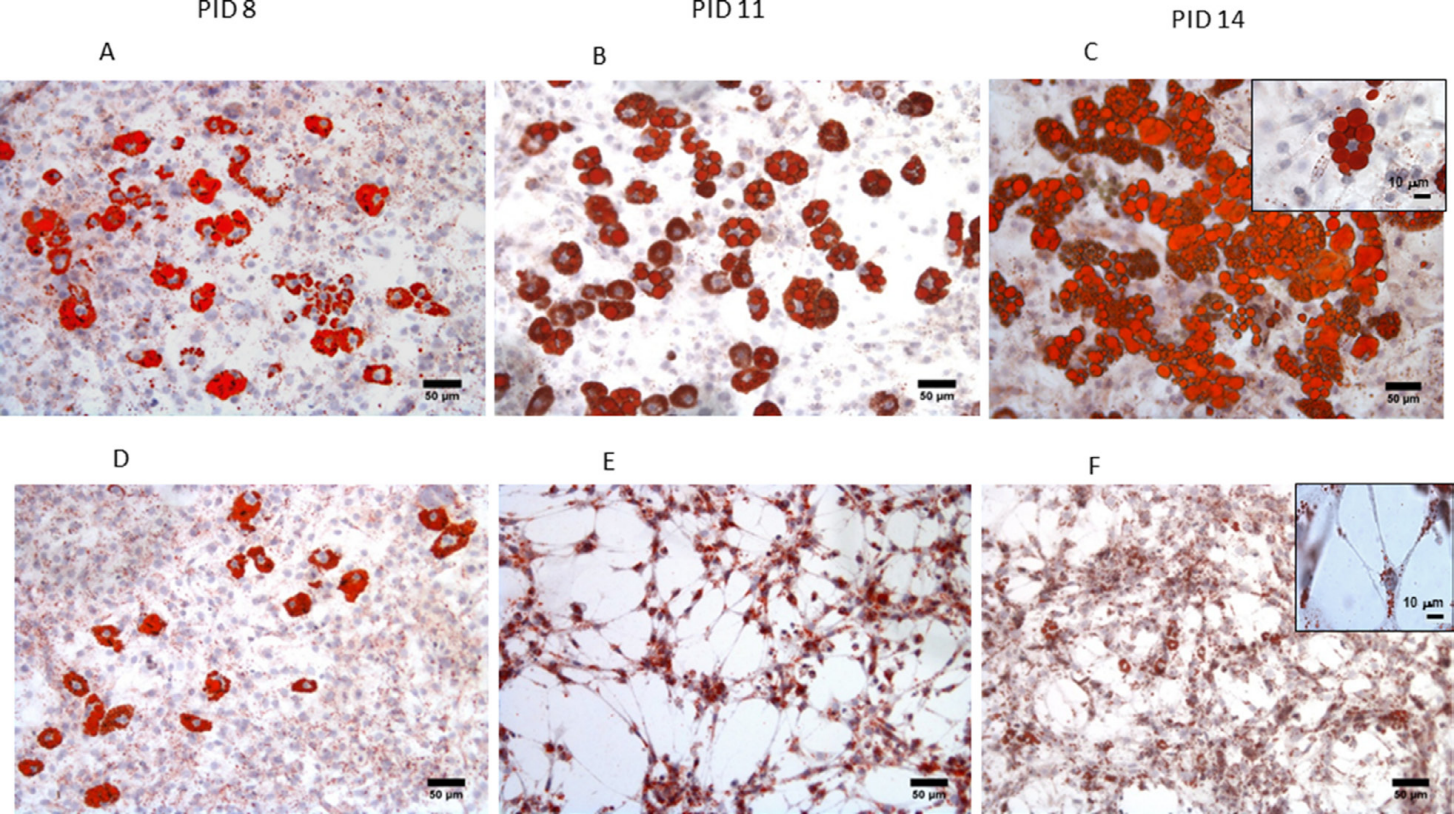
Morphological changes of 3T3-L1 adipocytes during co-culture with MiaPaCa2 cells Using Red Oil staining, we showed a progressive change in morphology of 3T3-L1 cells in co-culture with MiaPaCa2 cells (**D–F**) versus control adipocytes (**A–C**) from PID 8 to PID 14. (x100 magnification, scale 50 μm). Abbreviations: PID- post-induction day.

**Figure 2:**
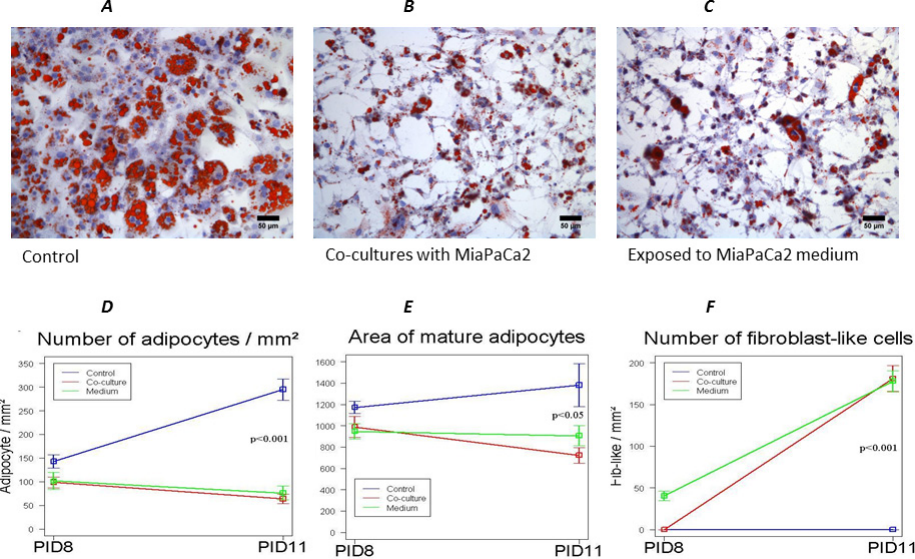
Effects of exposure to MiaPaCa2 cells conditioned medium on 3T3-L1 cells 3T3-L1 adipocytes exposed to MiaPaCa2 cell-conditioned medium still presented a fibroblast-like phenotype (**C**), similar to that previously observed in the co-culture system (**B**). Co-cultured adipocytes as well as adipocytes exposed to MiaPaCa2 medium, presented a progressive reduction in number (**D**) and area (**E**) when compared to control conditions. At the same time points, there was a progressive increase number of fibroblast-like cells in co-culture conditions as well as in adipocytes exposed to MiaPaCa2 medium, compared to controls (**F**). Data are presented as mean+standard error (m + SE). Abbreviations: PID- post-induction day.

After 6 and 9 days of 3T3-L1 adipocytes and MiaPaCa2 co-culture, we observed the abundant presence of fibroblast-like cells (Figure 1E, 1F and detail in Figure 1F), absent in control conditions (Figure 1B, 1C). During co-culturing, mature adipocytes progressively lost a considerable amount of lipid droplets, the nuclei became more centralized and the cells became elongated in shape, similar to a fibroblast morphology (Figure 1E, 1F). In particular, the number of fibroblast-like cells significantly increased at PID 11, after 6 days of co-culture, compared to control cultures of 3T3- L1 adipocytes (Figure 2F). This increased number of fibroblast-like cells was registered at the same time with a progressively decreasing number of mature adipocytes, which also presented a significant smaller diameter and area after 6 and 9 days of co-culture with MiaPaCa2 cells when compared with control 3T3-L1 mature adipocytes (Figure 2D, 2E).

In order to better understand the role played by tumor cells in the process of dedifferentiation we performed experiments using conditioned medium of MiaPaCa2 (CM-MPC) in cultures of 3T3-L1 adipocytes, starting at PID 5. 3T3-L1 exposed only to CM-MPC still dedifferentiated at PID 11, which presented a fibroblast-like phenotype, similar to that previously observed in the co-culture system (Figure 2C). However, at PID 11, adipocytes co-cultured with MiaPaCa2 cells were significantly smaller than adipocytes exposed only to CM-MPC (Figure 2E).

**Figure 3:**
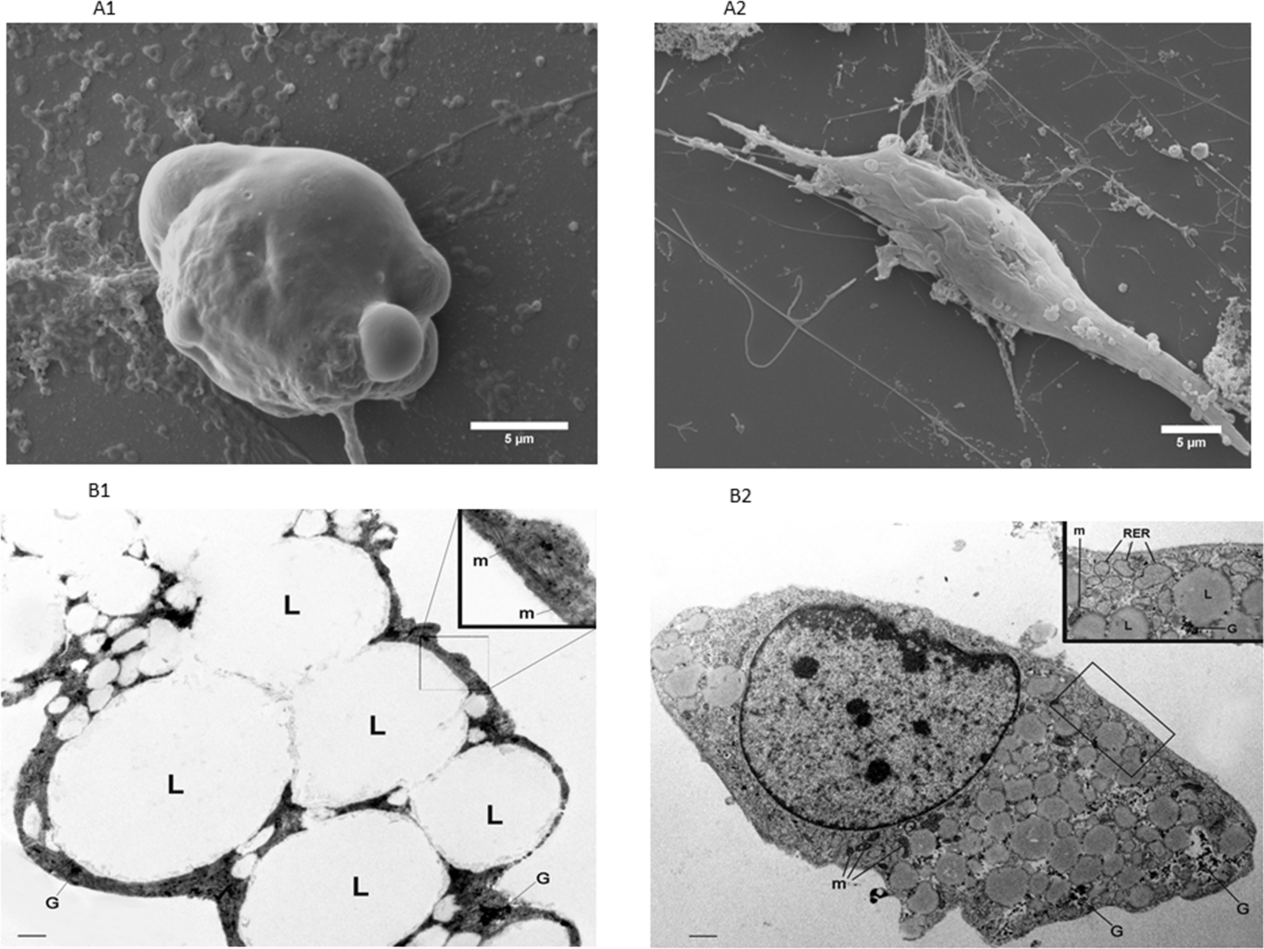
Representative electron microscopy images of 3T3-L1 adipocytes alone or in co-culture with MiaPaCa2 cells SEM images of 3T3-L1 adipocytes, after 6 days of co-culture with MiaPaCa2 cells (PID 11) showed a fibroblast-like morphology (**A2**), while the mature adipocytes, in control conditions, presented a typical spherical aspect (**A1**). Using TEM, we found that large lipid vacuoles occupied most of the cytoplasm of mature 3T3-L1 adipocytes at PID11 (**B1**); the insert represents the enlargement of the framed area showing typical elongated mitochondria with randomly oriented cristae. (scale bar: 0.3 μm; in insert scale bar: 0.9 μm). On the other hand, TEM of 3T3-L1 adipocyte co-cultured for 6 days with MiaPaCa2 cells (PID 11) presented visible and abundant dilated RER among small lipid vacuoles and glycogen (**B2**) (scale bar: 0.6 μm; in insert scale bar 1.2 μm). Abbreviations: PID- post-induction day, SEM - scansion electron microscope, TEM - transmission electron microscope, L - lipid vacuoles, G - Glycogen, RER - rough endoplasmic reticulum, m: mitochondria.

Electron microscopy of dedifferentiated adipocytes and controls is shown in Figure 3A and 3B. SEM clearly documented the changes in cell architecture after co-culture, with the appearance of small elongated cells with thin cytoplasmatic extensions (Figure 3A2), which were completely different from the typical spherical shape of the mature adipocyte (Figure 3A1).

Figure 3B1 and 3B2 show TEM images of representative cells in co-culture and control conditions. The morphology of cells in co-culture was completely different from the typical aspect of mature 3T3-L1 adipocytes which are characterized by large lipid droplets occupying almost all visible cytoplasm and typical elongated mitochondria with randomly oriented cristae (Figure 3B1). 3T3-L1 cells in co-culture presented most of the features typical of the early developing stages of stromal-vascular origin preadipocytes: i.e. cytoplasmic small lipid droplets and glycogen particles, but also features of active fibroblasts: i.e.: a well-represented and dilated rough endoplasmic reticulum (Figure 3B2).

### Dedifferentiated adipocytes lose the adipocyte-specific gene expression profile and acquire reprogramming gene expression

**Figure 4:**
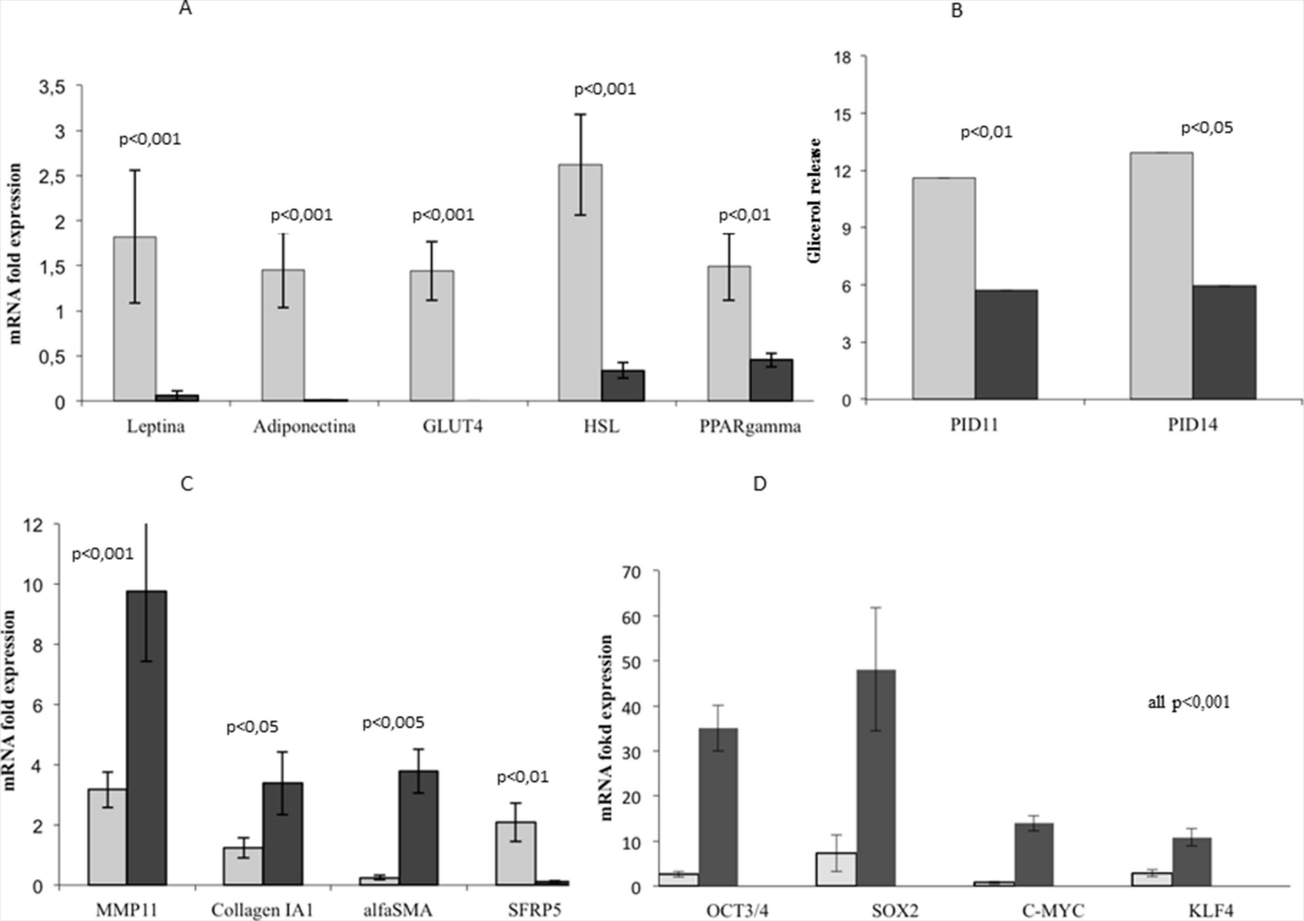
Functional changes of 3T3-L1 adipocytes in co-culture compared to control cells RT-PCR analysis showed that 3T3-L1 adipocytes co-cultured with MiaPaCa2 cells presented a significantly lower expression of mature adipose-specific genes after 9 days of co-culture with MiaPaCa2 cells (PID14), when compared to controls (**A**). At PID11 and 14, adipocytes co-cultured with MiaPaCa2 cells presented a decrease glycerol release in culture medium, compared to controls. (**B**). Additionally, 3T3-L1 adipocytes co-cultured with MiaPaCa2 cells presented a higher gene expression of fibroblast-specific markers, when compared to controls (**C**). After 9 days of co-culture (PID14), 3T3-L1 adipocytes had a higher expression of reprogramming genes, when compared with mature adipocytes (**D**). RT-PCR data are expressed considering as control, gene expression in 3T3-L1 at PID 8. Data are presented as mean+standard error (m + SE). Abbreviations: PID- post-induction day, RT-PCR- real time PCR.

In order to investigate the changes in the gene expression profile of dedifferentiated adipocytes, we analyzed, using RT-PCR, the expression of some mature adipocyte specific markers, such as leptin, adiponectin, GLUT4, HSL and PPARγ; moreover, we tested the expression of some fibroblast-specific genes, as MMP11, collagen I and α-SMA. After only 6 days of co-culture, we observed a significant decrease in some adipose markers expression compared to controls (Supplementary Figure 1A). After 9 days of co-culture with MiaPaCa2, adipocytes substantially lost leptin, adiponectin, GLUT4, HSL and PPARγ expression (Figure 4A). Moreover MMP11, collagen I and α-SMA expression significantly increased after 9 days of co-culture, further supporting a process of reprogramming/dedifferentiation of adipocytes toward fibroblast-like cells (Figure 4C).

To further characterize functional changes observed in 3T3-L1 adipocytes in co-culture, we measured glycerol release in culture medium after 6 and 9 days. Glycerol release significantly decreased in co-culture compared with control adipocytes at PID 11 and 14 (Figure 4B).

Finally, using RT-PCR assay, we analyzed the expression of genes required for the cell reprogramming process, such as Oct3/4, Klf4, c-Myc and Sox2. Our data showed a significant increase in the expression of all the genes considered important for reprogramming the mature cells genome, starting early at 3 and 6 days of co-culture with MiaPaCa2 (Supplementary Figure 1C). Moreover, after 9 days of co-culture, expression of Oct3/4, Klf4, c-Myc and Sox2 was dramatically increased in co-culture, compared to control mature adipocytes (Figure 4D).

### MiaPaCa2 cells express WNT5a

Many studies identified a crucial role for WNT pathways in oncogenesis, cancer progression and metastasis [17–21]. In PDAC, WNT5a is recurrently over-expressed and exerts a pro-oncogenic function, by promoting proliferation, migration and invasion of cancer cells [18]. However, opposite effects were obtained for WNT5a in other types of cancer [18]. Discrepant results could be dependent on the fact that the existence of two WNT5a isoforms, WNT5a-long (WNT5a-L) and WNT5a-short (WNT5a-S), exerting respectively anti- and pro-oncogenic effects [19] has been recently demonstrated.

**Figure 5:**
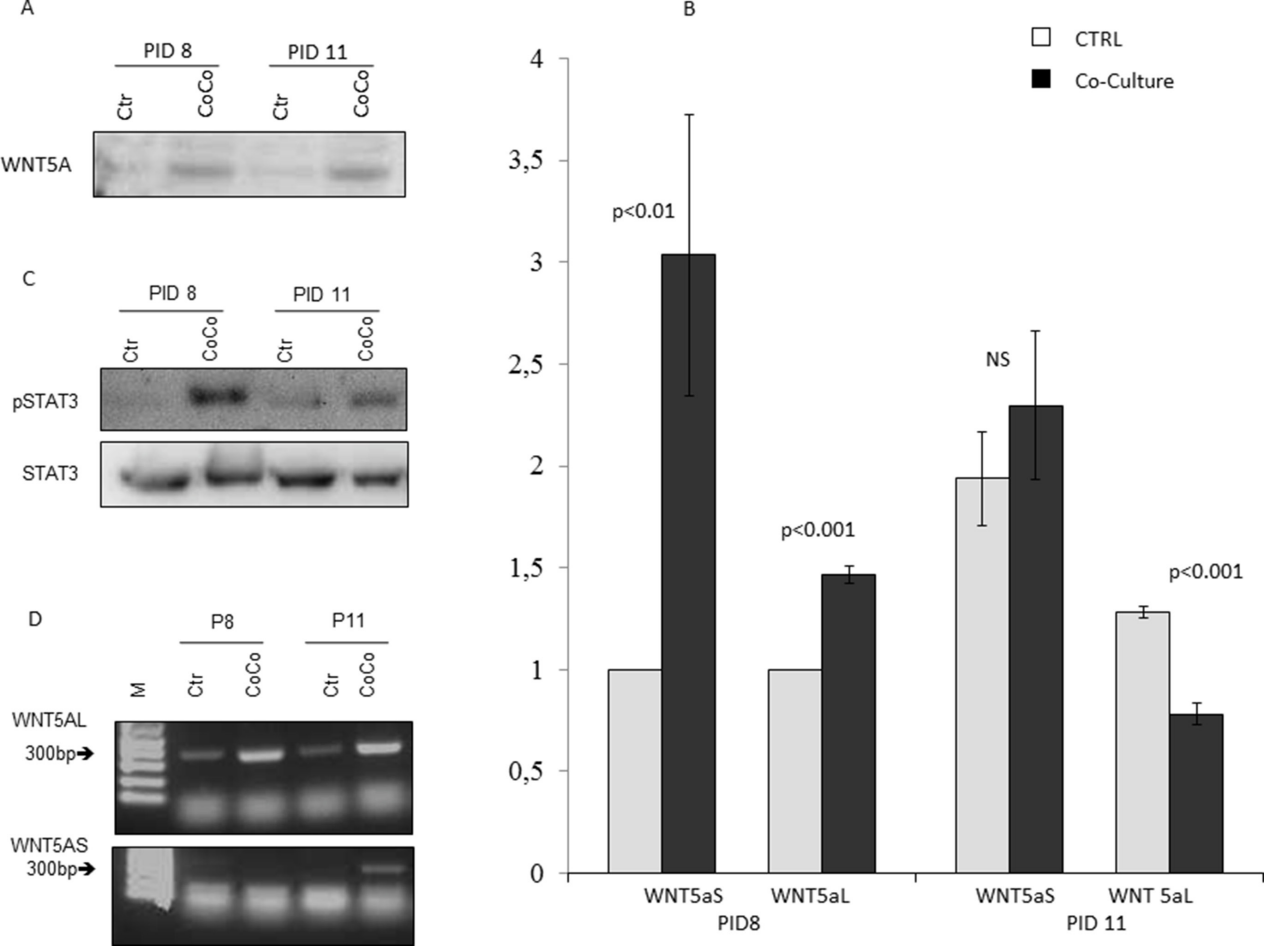
Activation of WNT5a pathway in MiaPaCa2 cells and adipocytes in co-culture WB analysis showed that the WNT5a protein expression in total cell extract of MiaPaCa2 was augmented after 3 (PID8) and 6 days (PID11) of co-culture (CoCo), when compared to control conditions (Ctr) (**A**). Additionally, RT-PCR analysis showed an increase expression of WNT5aS in MiaPaCa2 cells co-cultured with 3T3-L1 adipocytes, especially at PID 8 and a lower expression of WNT5aL (**B**). In order to investigate the pathway that might explain WNT5a up-regulation in our experimental conditions, we registered a higher expression by WB of STAT3 in total cell extract of MiaPaCa2 cells in co-culture with 3T3-L1 when compared to control conditions (**C**). Moreover, PCR analysis of WnT5aS and WNT5aL in 3T3-L1 alone (Ctr) and co-cultured (CoCo) with MiaPaCa2 showed an increased expression of WNT5aL in co-culture at PID 11 and 14, while WNT5a-S was not found in adipocytes maintained in control medium (**D**). RT-PCR data are expressed considering as control, gene expression in 3T3-L1 at PID 8, the results are presented as mean + standard error (m + SE). Abbreviations: WB- Western blot assay PID- post-induction day, RT-PCR – real time PCR; CoCo-co-culture condition; Ctr- control condition; WNT5aS-WNT5a short; WNT5aL- WNT5a long.

In our model WB analysis confirmed the expression of WNT5a in MiaPaCa2 cells and more abundantly in cells co-cultured with 3T3-L1 (Figure 5A). We then analyzed the expression of the two different WNT5a isoforms in MiaPaCa2 cells co-cultured with adipocytes or not (Figure 5B). We found a significant increase in WNT5a-S expression starting at an early stage of co-culture (PID8), and a significant decrease in WNT5a-L mRNA in more advanced phases (Figure 5B).

As it has already been shown that up-regulation of WNT5a could be done by STAT3 that recognizes a specific WNT5a promoter region, we investigated the phosphorylation of STAT3 by WB. Our data confirmed that MiaPaCa2 cell co-cultivated with 3T3-L1 presented an increased activation of STAT3 compared to controls conditions, starting at PID8 (Figure 5C).

Finally as WNT5a inhibition is crucial in promoting differentiation of mesenchymal stem cells into preadipocytes [29], we investigated its expression also in 3T3-L1 adipocytes alone and after co-culture with MiaPaCa2 cells. We found more pronounced expression of WNT5a-L after co-culture with pancreatic cancer cells (Figure 5D). The levels of mouse WNT5a in the medium was significantly higher in co-cultures compared to controls at the same stage of maturation (data not shown in figures).

### The dedifferentiation of adipocytes, induced by pancreatic cancer cells, is associated with an activation of the WNT5a-dependent signaling pathways

WNT5a is a highly conserved lipid modified glycoprotein belonging to the WNT family and a member of the non-canonical beta-catenin independent pathway [30, 31]. In mesenchymal stem cells it has been demonstrated that WNT5a signaling is mediated by JNK-dependent non-canonical pathway with a consequent activation of c-Jun and AP-1 [19], transcription factors known to regulate cell proliferation, differentiation and survival, as well as tumor promotion and progression [18].

**Figure 6:**
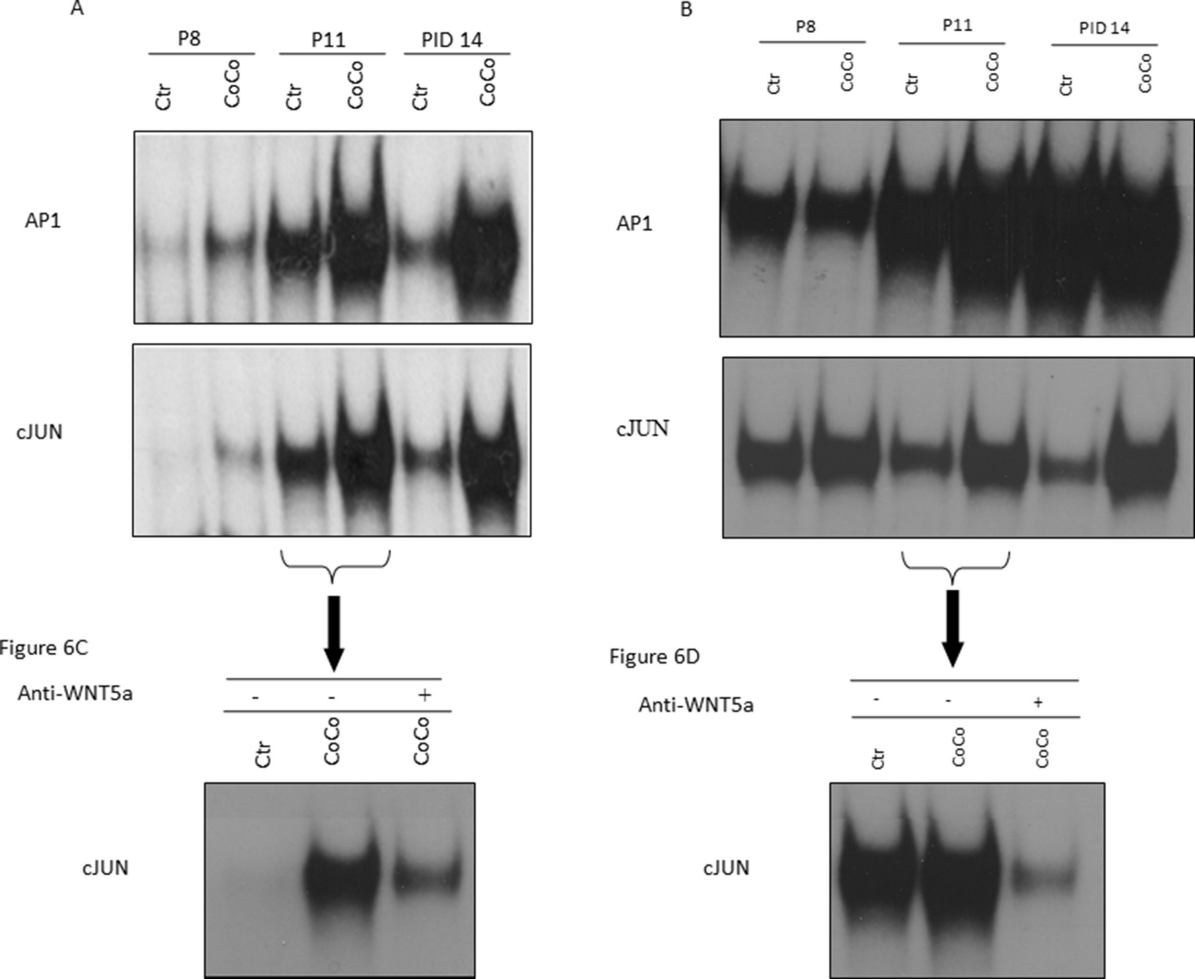
Inhibition of the WNT5a pathway may control the adipocytes' phenotype shift In order to investigate the pathway that might be activated by WNT5a up-regulation in our experimental setting, expression of activated c-Jun and AP1 in 3T3-L1 adipocytes (**A**) and MiaPaCa2 cells (**B**) at PID8, PID11 and PID14 was examined by EMSA. As the c-Jun expression appeared augmented in co-culture at PID11, we treated it for 24h with a WNT5a neutralizing antibody. Our EMSA results proved that c-Jun activation was inhibited by this treatment in 3T3-L1 adipocytes (**C**) and MiaPaCa2 cells (**D**). Abbreviations: PID- post-induction day, EMSA- Electrophoretic Mobility Shift Assay, AP-1: activator protein-1.

We performed EMSA experiments, using nuclear protein extracts of 3T3-L1 grown in control medium or in co-culture at different time points (Figure 6). Nuclear extracts from 3T3L1 co-cultured cells, at PID 11/14, presented an increased binding capacity on both c-Jun and AP-1 binding sites, two WNT5a-dependent signaling pathways (Figure 6A).

Moreover, EMSA of nuclear extract of MiaPaCa2 cells showed an increased binding of c-Jun and AP1 after co-culture with adipocytes (Figure 6B). So we hypothesized that WNT5a may also have an autocrine and paracrine function on MiaPaCa2 cells, probably mediated by STAT3 activation.

To further support the WNT5a role in adipocytes dedifferentiation phenomena, we blocked the WNT5a pathway by adding an anti-WNT5a antibody in the culture medium (Figure 6C, 6D). Interestingly, in the presence of the neutralizing antibody, 3T3-L1 adipocytes co-cultured with MiaPaCa2 presented a decreased activation of both c-JUN and AP1 transcriptional factors when compared to co-cultured conditions in the absence of antibody (Figure 6C, 6D).

### Inhibition of WNT5a signal revert the fibroblast-like phenotype of 3T3-L1 adipocytes co-cultured with MiaPaCa2 cells

We first studied the expression in our experiment of the WNT5a antagonist Secreted frizzled-related protein 5 (SFRP-5). SFRP-5 is a highly conserved secreted protein which inhibits WNT-5a signaling by competing for the binding with its receptor [31, 32].

In our co-culture at PID 11 and 14, SFRP-5 expression in 3T3-L1 adipocytes was almost completely suppressed compared to controls (Figure 4 and Supplementary Figure 1).

In the paper of Zhe et al. [32], it was demonstrated that treatment with SFRP-5 was as efficient in blocking the WNT-5a pathway as WNT-5a silencing.

**Figure 7:**
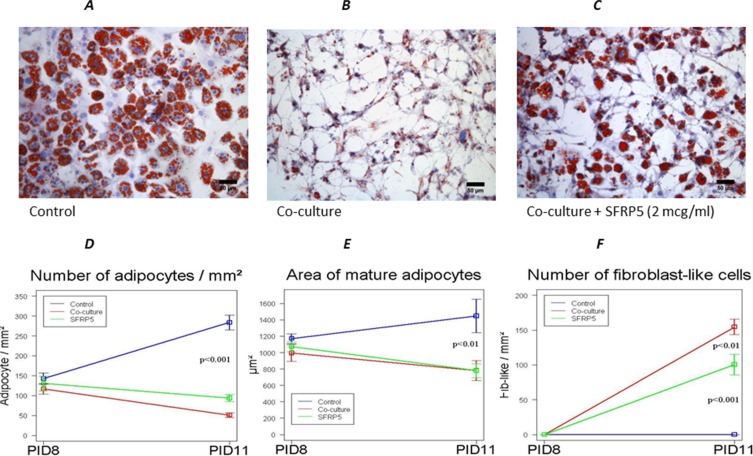
Inhibition of WNT5a signal prevents 3T3-L1 dedifferentiation into fibroblast-like cells after co-cultured with MiaPaCa2 cells Effect of SFRP-5 addition to the medium on the dedifferentiation of adipocytes in co-culture (**C**). Number and area of adipocytes (**D, E**) as well as number of fibrobast-like cells in co-culture with SFRP5 (**F**) compared to co-culture alone. Data are presented as mean+standard error (m + SE). Abbreviations: PID- post-induction day, SFRP5-secreted frizzled related-protein 5.

We repeated the experiment adding increasing doses (0,5 and 2 μM/ml) of recombinant SFRP-5, starting at PID 8 and then every 2 days to the co-culture medium. We found that SFRP-5 at the highest dose was able to block the dedifferentiation of adipocytes in co-culture (Figure 7C). The number of fibrobast-like cells in co-culture with SFRP5 was significantly lower than in the co-culture alone and the number of adipocytes significantly higher (Figure 7D, 7F).

## DISCUSSION

**Figure 8:**
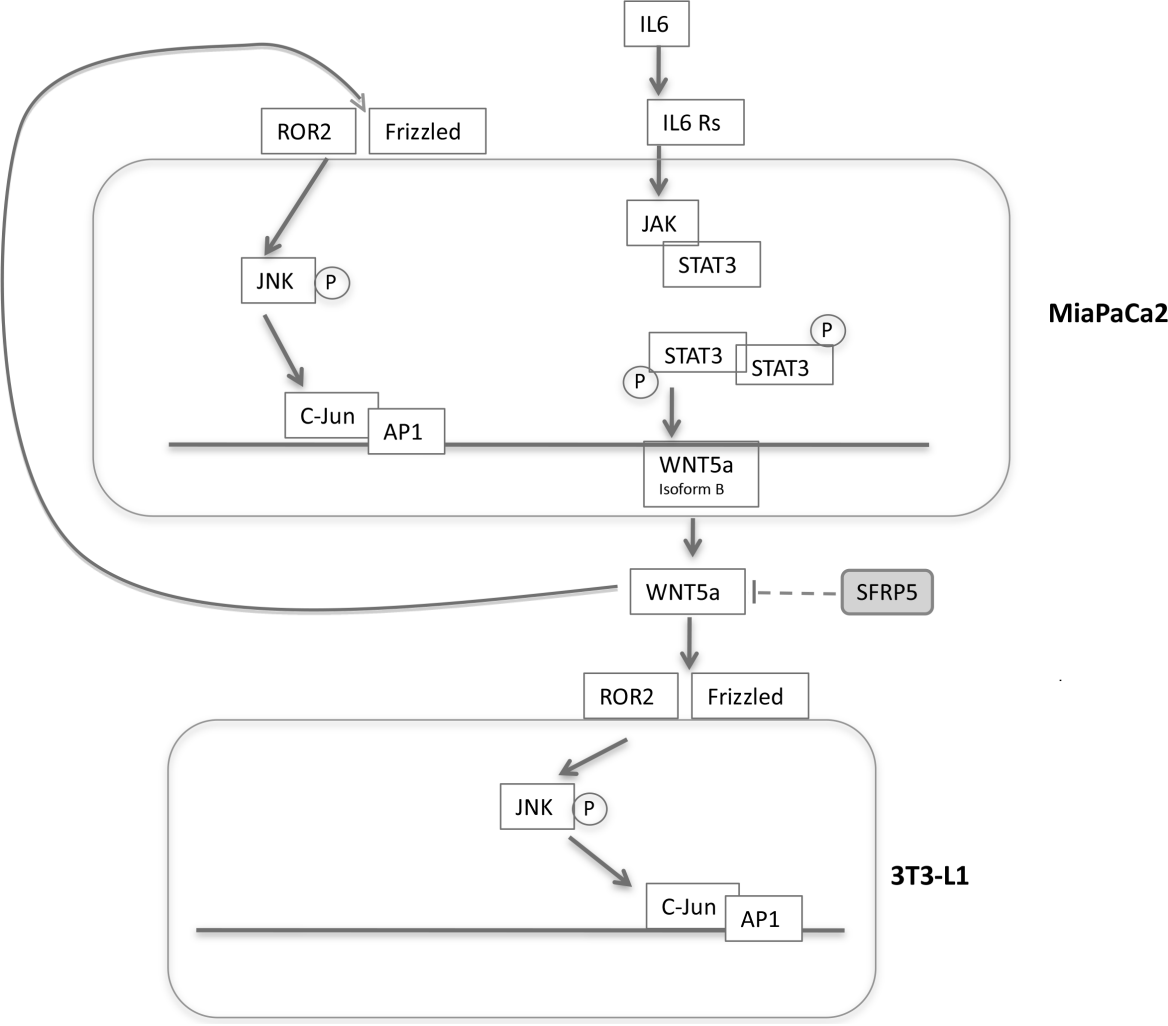
Mechanisms hypothesized in the crosstalk between pancreatic cancer cells and adipocytes Adipocytes in pro-inflammatory conditions produce high levels of IL6, that could be a potential regulator of WNT5a expression and secretion in MiaPaCa2 through IL6-STAT3 signaling pathway. The secretion of WNT5a protein is associated to an increased signal of c-Jun and AP1 in 3T3-L1 after co-culture, representing one of the pathways activated by WNT5a up-regulation. The activation of WNT5a receptor and downstream signaling is prevented by the binding with its inhibitor SFRP5. Abbreviations: IL6- interleukine 6, AP-1- activator protein-1, SFRP5-secreted frizzled related-protein 5.

In this study we described in an experimental model of pancreatic cancer, reprogramming/dedifferentiation of white adipocytes into fibroblast-like cells, reproducing, *in vitro*, phenomena that could contribute to the profound changes observed in tumor microenvironment and cancer development and progression. 3T3-L1 adipocytes in co- culture expressed reprogramming genes and changed their phenotype in favor of that of fibroblast-like cells, with characteristic electron microscopic morphology [33]. In *in vitro* and *in vivo* models of breast cancer it has been recently demonstrated that peritumoral adipocytes can undergo dedifferentiation phenomena first into cancer associated adipocytes (CAA) and then into CAF, contributing to the desmoplastic reaction surrounding the tumor [34–36]. This evidence led some authors to hypothesize a relevant role for AT not only meeting the increased metabolic requests of proliferating tumor cells but also in matrix remodeling and cancer progression. This could be possible since adipocytes have been shown to dedifferentiate into immature cells with characteristics similar to mesenchymal cells in different experimental settings [34]. In line with this hypothesis our data support the existence of a process characterized by adipocytes dedifferentiation/reprogramming toward fibroblasts-like cells, first described after co-culture with pancreatic cancer cells. In our experiment it could be hypothesized that increased expression of MMP-11 and Collagen I found after co-culture, may impact cancer microenvironment, to explain the tumor-promoting effect of dedifferentiated cells observed in other experimental settings [37]. However the mechanisms contributing to this dedifferentiation process are not completely understood. It has been reported that circulating concentration of WNT5a are increased in human obesity [17] and that noncanonical Wnt signaling is activated, probably contributing to the pro-inflammatory state in visceral adipose tissue and to the development of obesity-associated co-morbidities. WNT5a has also been reported to be up-regulated in different types of cancer, especially in human PDAC [18] and represents a key cascade molecule in Extracellular Matrix remodeling [34]. In line with the literature, in our study we show that co-culture with adipocytes increased WNT5a protein levels by MiaPaCa2 cells. We found significant increase in WNT5aS expression starting early after co-culture of MiaPaca2 cells with adipocytes. It is important to note that WNT5aS contain sequences recognized by NF-kB and STAT3 [30]. Adipocytes in pro-inflammatory conditions produce high levels of IL6 that could be a potential regulator of WNT5a expression through STAT3 signaling pathway. In our system, due to the crosstalk between adipocytes and pancreatic cancer cells, we observed an increase in STAT3 phosphorylation in Tyr-705 in cancer cells; as a consequence of IL6/STAT3 signaling pathway activation we observed in MiaPaCa2 cells an increase in WNT5a expression (Figure 8). Afterwards, we analyzed the activation of c-JUN transcriptional factor DNA-binding via WNT5a/Ror2 signaling. All the results of our experiment confirmed that WNT5a is active after co-culture with pancreatic cancer cells and has an autocrine and paracrine function, activating the c-JUN/AP1 pathway also in 3T3-L1 (Figure 8). As a consequence of this activation in adipocytes, we also observed the increased expression of proteins deeply involved in the WNT5a pathway, such as MMP, which with other proteins are responsible for stroma remodeling.

Globally our data seem to point to the activation of WNT5a as a crucial factor in mediating dedifferentiation of adipocytes. In fact, treatment with antibody against WNT5a reverted the changes in intracellular signaling observed after co-culture in adipocytes. Moreover increasing doses of recombinant SFRP-5 were able to block the dedifferentiation of adipocytes in co-culture.

Taken together, the findings of the present study support a model in which pancreatic cancer cells may reprogram adipocytes to fibroblast-like cells. Reprogrammed adipocytes produce growth-promoting cytokines and provide lipids and other metabolites to cancer cells, which promotes uncontrolled tumor growth. Cancer cells may induce up-regulation of different adipocyte-secreted factors as TGF-beta, TNF-alpha and MMP. Activated adipocytes after exposure to these factors may dedifferentiate into preadipocyte/fibroblast-like cells, which in turn sustain cancer cell growth and invasion.

Further studies seem necessary to confirm these findings also in co-cultures using human adipocytes as well as *in vivo* models and to find new molecular targets that may influence tumor indirectly through changes to its microenvironment, which could represent a new target for cancer treatment. This crosstalk has been studied only in some cancer models where AT is strictly and anatomically in contact with cancer cells, as in breast cancer, but not yet extensively in solid tumors, such as pancreatic cancer, where AT has been regarded only as a generic risk factor. The plasticity of AT and the existence of dedifferentiation phenomena could thus bring new light into the complex relation between obesity, AT dysfunction and increased cancer risk.

## MATERIALS AND METHODS

### Cell culture

MiaPaCa2 (ATCC) and 3T3-L1 cells (ECACC Sigma-Aldrich) were cultured at 37°C in 5% CO2 respectively in RPMI1640 and DMEM/GlutaMAX culture medium (Gibco), with both containing FBS 10% and 1% Antibiotic Antimycotic Solution (SIGMA). At 85–90% of confluence, 3T3-L1 cells were detached by trypsin-EDTA (Gibco) and seeded in 6-wells (Becton Dickinson) containing a presterilized slide (Menzel-glaser Thermo Scientific). At 90% of confluence, cells were induced to differentiate in DMEM/F12 containing 10% FBS, 1% Antibiotic Antimycotic Solution, 0.2 mM IBMX, 10 μM rosiglitazone, 1 μM dexamethasone, 10 μg/ml insulin for 3 days. After 72 h post-induction day (PID 3) medium was replaced with an adipocyte maintaining medium (AMM), composed of DMEM/F12 enriched with 10% FBS, 1% Antibiotic Antimycotic Solution, 10 μg/ml insulin (Sigma), in which cells were cultured for 2 days. Five days after adipocyte induction, the cells were co- cultured with MiaPaCa2 using a transwell culture system (0,4 μm pore size; Becton Dickinson) in AMM without insulin. 15,000 cells/cm^*2*^ of MiaPaCa2 cells were seeded in the top chamber. Co-culture medium was replaced every three days at 50%, and cells were co-cultured with MiaPaCa2 cells for further 3 days (PID8), 6 days (PID11) and 9 days (PID14); MiaPaCa2 and 3T3-L1 adipocytes were cultivated alone as controls and evaluated at the same time points. Cell viability was determined by trypan blue staining and viable cells were counted using the Countess Automated Cell Counter (Invitrogen).

We repeated our experiment using conditioned medium of MiaPaCa2 cells (CM-MPC) in culture of 3T3-L1 alone. 3T3-L1 cells were cultured starting from PID 5 with 50% of CM-MPC and 50% of AMM without insulin. Every 3 days 50% of 3T3-L1 culture medium was replenished with fresh CM-MPC and AMM.

In the experiment to inhibit adipocyte dedifferentiation, 1 μg of anti-WNT5a antibodies (sc-23698, Santa Cruz Biotechnology), 2 μg of non specific antibody of the same subclass were added per well. Moreover increasing doses (0,5 and 2 uM/ml) of human recombinant SFRP5 (R & D Systems Catalog Number: 6266-SF), were added to the co-culture medium, starting at PID 8 and then every 2 days.

### Oil Red O staining and optical image capturing and analyses

After washing with phosphate buffer solphate (PBS) 0.1M pH 7.4, cell cultures were fixed for 20 min with 10% neutral buffered formalin. Cells were washed with sterile double distilled water and subsequently with 60% isopropanol for 2 minutes (min) and stained with a filtered 0.35% Oil Red O solution in 60% isopropanol for 10 min at room temperature. Then, cells were washed with sterile double-distilled water and stained with Mayer's Hematoxylin (Bio-Optica) ready-to-use solution for 1 min at room temperature and then washed again with sterile double-distilled water. Slides were treated with Dako Faramount Aqueous Mounting Medium.

Cells were observed in an Olympus BX51 photomicroscope equipped with a KY-F58 CCD camera (JVC). The images were analyzed using ImageJ software 1.47e version to count the cells within 10 representative fields (100x magnification, number of cells expressed on mm²) and to calculate the area of 10 randomly chosen cells (400x magnification, area expressed in μm²).

### Scanning electron microscopy (SEM)

3T3-L1 cells differentiated to adipocytes for 11 days (PID11 control) and 3T3-L1 cells co-cultured with MiaPaCa2 cells (PID 11) were grown on glass cover slips for 6 days (PID11 co-culture), washed with PBS pH 7.4 0.1 M and then dehydrated with alcohols of increasing concentration (30°, 50°, 80°, 95° and 100°, 5 minutes for each step), dried, and fixed on stubs with adhesive colloidal silver and metallizer MED 010 (Balzers) and observed under an electron microscope XL30 ESEM (FEI-Philips).

### Transmission electron microscopy (TEM)

After co-culture, 3T3-L1 as well as dedifferentiated adipocytes, at PID 11 and 14, were fixed in 2% glutaraldehyde in 0.1 M phosphate buffer (pH 7.4) for 1 hour, post-fixed in a solution of 1% osmium tetroxide and 1% potassium hexacyanoferrate (II), dehydrated in ethanol and finally epoxy-resin embedded. Semi-thin sections (2 μm) were stained with toluidine blue. Thin sections obtained with an MT-X ultratome (RCM, Tucson, AZ) were mounted on copper grids, stained with lead citrate and examined with a CM10 transmission electron microscope (Philips, Eindhoven, The Netherlands).

### RNA extraction, cDNA synthesis and Real-time polymerase chain reaction (RT- PCR)

RNA was extracted from 600 μl of cellular lysate, following QIAGEN's RNeasy Mini Kit^®^ protocol for QIAcube automatic extraction. Extracted and purified RNA was checked with an Agilent RNA 6000 Nano Kit on an RNA Nanochip for Agilent 2100 bioanalyzer. Reverse transcription of RNA to cDNA was performed with iScript™ cDNA Synthesis Kit by Bio-Rad using the following protocol: 5 min 25°C, 30 min 42°C, 5 min 85°C. Real-time PCR (RT-PCR) was performed with a Bio-Rad MyiQ^™^ RT-PCR System, using a QuantiTect™ SYBR^®^ Green RT-PCR Kit by QIAGEN, according to the manufacturer's protocol. Briefly, PCR was performed in a final volume of 25 μl, including 100 ng of cDNA, 12,5 μl of QuantiTect™ SYBR^®^ Green PCR Master Mix, 7,5 μl of RNAse free water and 2,5 μl of QuantiTect^®^ Primer Assay. RNA was denaturated at 95°C for 15 minutes, while amplification was performed for 40 cycles under the following conditions: 15 s at 94°C, 30 s at 55°C and 30 s at 72°C, with QuantiTect™ Primers Assays (from QIAGEN^®^) for leptin, adiponectin, GLUT4, HSL, collagen I A1, MMP11, α-SMA, SFRP5, Klf4, c-Myc, Oct3/4, Sox2. RT-PCR data were analyzed with Bio-Rad iQ™ Optical System Software. Expression levels were normalized using internal control (β-Actin). Relative expression levels were calculated using the formula 2^−(ΔΔCt)^. Each analysis was conducted at least in triplicate.

It has been recently shown that WNT5a encodes two isoforms with distinct functions in cancers [19, 30]. We used sequences published in a previous work [19], purchased from Eurofins Genomics. cDNA was denatured at 95°C for 15 minutes, while amplification was performed for 40 cycles under the following conditions: 15 s at 94°C, 30 s at 58°C and 30 s at 72°C.

### Lipolysis assay

A cultured adipocytes lipolysis assay kit (Glycerol cell based Assay kit. Cat 10011725, Cayman) was used to determine free glycerol released in medium during co- culture at PID 11 and 14. Absorbance was measured at 540 nm. A standard curve was created and the amount of free glycerol was determined in duplicate.

### Western blot

MiaPaCa2 cells were lysed in ice-cold buffer containing 20 mM HEPES, pH 7.4, 420 mM NaCl, 1 mM EDTA, 1 mM EGTA, 1% Nonidet-P40 (NP- 40), 20% glycerol, protease cocktail inhibitors (GE Healthcare, Amersham Place, UK) and phosphatase cocktail inhibitors. The lysates were centrifuged at 25,000 g for 30 min. 3T3-L1 cells were lysed in ice-cold lysis buffer containing 50 mM Hepes (pH 7.4), 5 mM EDTA, 50 mM NaCl, 1% Triton X-100, 50 mM NaF, protease and phosphatase cocktail inhibitors and the lysates were centrifuged at 12,000 xg for 15 min. Supernatant of lysates of both type cells were collected and their protein concentrations were determined by the method described by Bradford (1976). Equal amounts of protein (40 μg total protein/lane) were loaded on 10% SDS- polyacrylamide gels. Electrophoresis was performed at 100 V with a running buffer containing 0.25 M TrisHCl, pH 8.3, 1.92 M glycine, and 1% SDS. The resolved protein were electroblotted onto a PVDF membrane (Immobilon P, Millipore, Bedford MA). The membranes were blocked with 5% non-fat dried milk in TBS-T (TBS containing 0.1% (v/v) Tween 20) and subsequently incubated overnight at 4°C with the primary antibodies (WNT5a, Santa Cruz biotecnology; pSTAT3(Y705) and totalSTAT3 (Cell Signaling). After washing, the membrane was further incubated with a HRP-conjugated secondary antibody (anti-goat, anti-rabbit, Santa Cruz biotecnology) for 1.5 h and, after washing, the membrane was developed using chemiluminescent detection system (Immun-StarTM WesternCTM Kit, Bio-Rad, Hercules, CA). Blotted proteins were detected using the ChemiDoc XRS Imaging System (Bio-Rad, Hercules, CA). After stripping, membranes were re-hybridized with the correspondent antibodies.

### ELISA

Mouse Wnt-5a in culture medium was measured in the different experimental conditions using a Mouse Protein Wnt-5a ELISA Kit (CUSABIO Catalog Number. CSB-EL026138MO). ELISA assays were done in 2 different sets of experiments and each experimental point was analyzed in duplicate. (Sensitivity: 0,78 pg/ml; excellent specificity for detection of mouse WNT5a, no significant cross-reactivity).

### Electrophoretic mobility shift assay

Nuclear extracts of MiaPaCa2 and 3T3-L1 cells were prepared as previously described [38]. DNA binding reaction consisted of 4 μg of nuclear extract incubated with 105 cpm of 32P-labeled double-stranded oligonucleotides containing the consensus c-JUN DNA binding site (5′-GGGCTTGATGAGTCAGCCGGACC-3′) or AP1 DNA binding site (5′-CGCTTGATGACT CAGCCGGAA-3′), in a final volume of 15 μl buffer (20 mM Hepes, pH 7.9, 50 mM KCl, 0.5 mM dithiothreitol, 0.1 mM EDTA, 2 mg of poly(dI-dC), and 10% glycerol). Products were fractionated on a non-denaturing 5% polyacrylamide gel. The gel was dried and auto-radiographed. Specificity of the retarded bands was demonstrated by competition with 100-fold excess of specific unlabeled oligonucleotide (not shown).

### Statistical analysis

Data were presented as mean+standard error (m + SE) in the different experimental conditions. Data were analysed for their statistical significance after evaluating their normal distribution. Differences in mRNA expression were analyzed using the ANOVA test with the least significant difference (LSD) post-hoc test to detect statistical differences between the different experimental conditions. Differences were considered statistically significant at *p* < 0.05. Statistical analysis was performed by using SPSS Statistical software (Statistical Package for the Social Science, version 21 for Windows).

## SUPPLEMENTARY MATERIALS FIGURE


