# Supplementary material for: Adipocytes WNT5a mediated dedifferentiation: a possible target in pancreatic cancer microenvironment
Source: Oncotarget. 2016 Mar 6;7(15):20223–35. doi: 10.18632/oncotarget.7936 (PMC4991449; doi:10.18632/oncotarget.7936)
Supplement: Supplementary file 1 [file oncotarget-07-20223-s001.pdf]

Adipocytes WNT5a mediated dedifferentiation: a possible target in pancreatic cancer microenvironment

Supplementary Materials

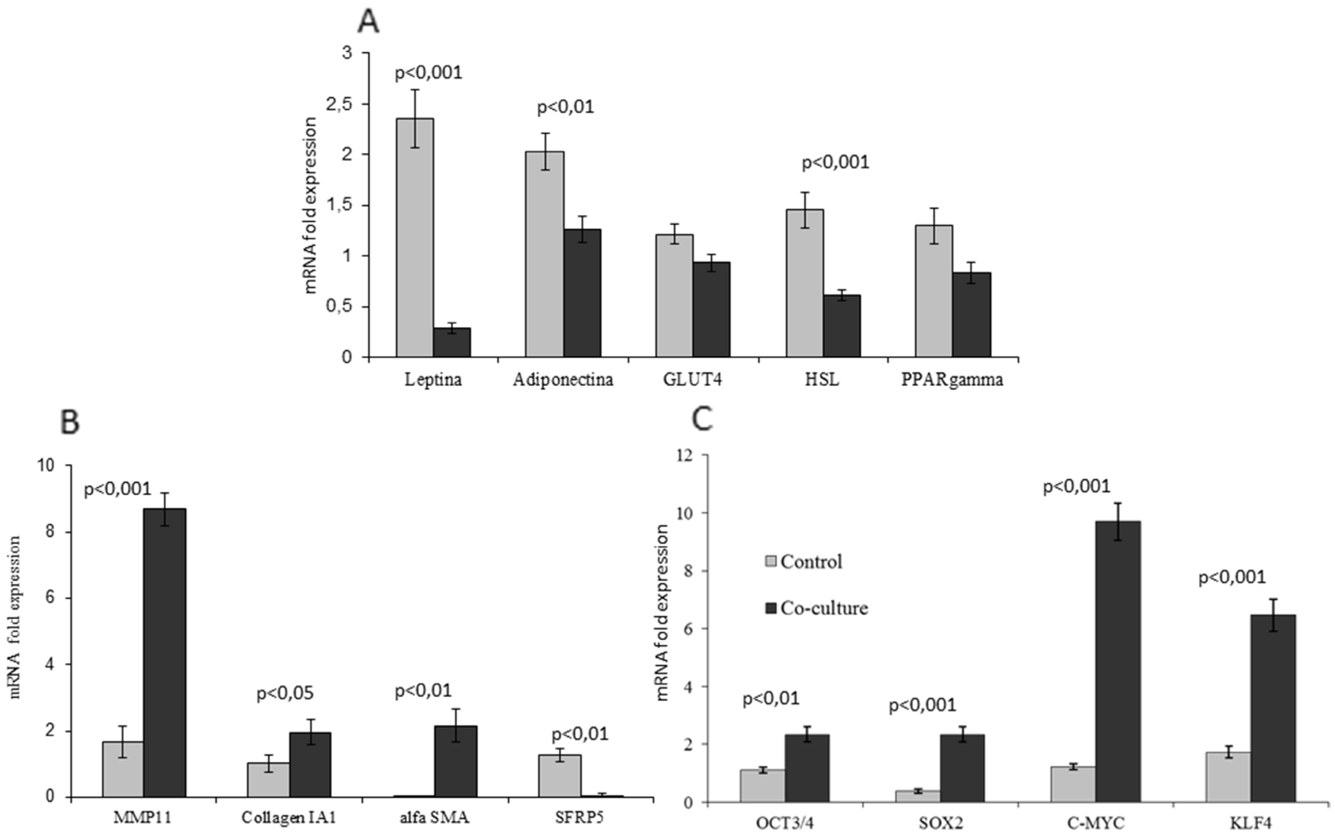

Supplementary Figure S1: Functional changes in 3T3-L1 adipocytes after 6 days of co-culture, compared to control conditions. RT-PCR analysis showed that 3T3-L1 adipocytes co-cultured with MiaPaCa2 cells presented a significantly lower expression of mature adipose-specific genes after 6 days of co-culture with MiaPaCa2 cells (PID11), when compared with controls (**A**). Additionally, at the same time-point, 3T3-L1 adipocytes co-cultured with MiaPaCa2 cells presented a higher gene expression of fibroblast-specific markers, when compared with controls (**B**). Equally, after 6 days of co-culture (PID11), 3T3-L1 adipocytes had a higher expression of reprogramming genes, when compared with mature adipocytes (**C**). RT-PCR data are expressed considering as control gene expression in 3T3-L1 at PID 8, then the results are presented as mean + standard error (m + SE). Abbreviations: PID- post-induction day, RT-PCR- real time PCR.
